# Pioneering Studies on Cephalopod's Eye and Vision at the Stazione Zoologica Anton Dohrn (1883-1977)

**DOI:** 10.3389/fphys.2016.00618

**Published:** 2016-12-23

**Authors:** Ariane Dröscher

**Affiliations:** Department of Letters and Philosophy, University of TrentoTrento, Italy

**Keywords:** cephalopod vision, history of vision research, Karl von Frisch, Carl von Hess, Zoological Station Anton Dohrn, color discrimination, history of experimentalism

## Abstract

From the late nineteenth century onwards, the phenomena of vision and the anatomy and physiology of the eye of marine animals induced many zoologists, ethologists, physiologists, anatomists, biochemists, and ophthalmologists to travel to the Zoological Station in Naples. Initially, their preferred research objects were fish, but it soon became evident that cephalopods have features which make them particularly suited to research. After the first studies, which outlined the anatomical structure of cephalopods' eyes and optic nerves, the research rapidly shifted to the electrophysiology and biochemistry of vision. In the twentieth century these results were integrated with behavioral tests and training techniques. Between 1909 and 1913 also the well-known debate on color vision between ophthalmologist Carl von Hess and zoologist Karl von Frisch took place in Naples. Largely unknown is that the debate also concerned cephalopods. A comparative historical analysis of these studies shows how different experimental devices, theoretical frameworks, and personal factors gave rise to two diametrically opposing views.

## Introduction

Of all the senses, visual perception has received by far the greatest attention. The main reason is that our human encounter and exchange with the environment mostly relies on optic stimuli. Another reason is that humans usually look into each other's eyes in order to access the other's emotional and mental sphere. In the twelfth century, Hildegard von Bingen expressed this desire with the aphorism “The eyes are the windows of the soul.” The considerable advancement of notions and techniques of sensory physiology in the second half of the nineteenth century raised expectations that it might be possible to penetrate also the minds of animals. Excited by John Lubbock's book *On the senses, instincts and intelligence of animals with special reference to insects* (Lubbock, 1888), on 3 January 1892 Baron Farrer wrote to Lubbock from Naples: “it is clear that the thing now to do is to try to find out, as you have done, what animals really do see, hear and feel, rather than what their organs ought to enable them to do. What a world of possibilities the subject opens to us” (quoted from Hutchinson, 2014, p. I, 322).

From the first decades of the twentieth century cephalopods became a favorite object of vision research. Probably no other invertebrate depends so heavily on visual information. Vision is indispensable for their moving and hunting, as well as for their diurnal rhythm and the correct functioning of their hormonal glands (Wells and Wells, 1959; Wells, 1960). Although cephalopod eyes are significantly distinguished from vertebrate eyes, they have also important analogies (Ogura et al., 2004), and they are particularly suited to vision research. Fröhlich (1913a) already listed the following favorable features: (1) the eye is of large size; (2) it survives long after its extirpation and (3) has only one type of receptor cell; (4) the optic nerves route directly behind the photocells; (5) the optic nerves are very long (in *Octopus*: 18 mm) and end in a separate part of the nervous system posterior to the eyeball, the optic lobe. In addition, (6) cephalopods are well suited to learning experiments and conditioning.

In the second half of the nineteenth century still little was known about the life of cephalopods. Matters changed with the creation of marine biology stations, first the *Stazione Zoologica Anton Dohrn* in Naples (1872), the two French *Station biologique* of Roscoff (1872) and the *Station marine* of Wimereux (1874), and then many others. Most of these marine stations had the two-fold purpose of (i) promoting knowledge about marine animals and (ii) “renaturalizing” biological research, which had become increasingly confined to urban laboratories. Yet whereas the French stations largely maintained their “field station” nature, Dohrn's *Stazione* developed into a research institution at which many important laboratory techniques were devised (Bont, 2014). Moreover, because the Station was an international research facility, it hosted scholars from many different countries and working in almost all the biomedical disciplines. This greatly favored interdisciplinary exchange; yet it made the Neapolitan research output very heterogeneous and, as we shall see, it sometimes led to conflicting research projects being carried out simultaneously at the same site.

## Pioneering studies on the anatomy and physiology of the cephalopod eye

The first studies on cephalopods carried out at the Stazione Zoologica provided the basic knowledge on the anatomy, physiology, development, habitat and phylogeny of these then still mysterious animals. They culminated in the two fundamental works by Jatta (1896) and Naef (1923)^1^. Quite soon, however, some very special features of this animal group became evident and led to the development of specific laboratory techniques. One of them concerned the visual organ.

Due to its large size and relatively simple anatomical structure, the retina of cephalopods was soon appreciated for comparative studies and as a model for the photoreceptive mechanism. As early as 1884, Hermann Grenacher showed that the octopus retina, despite its superficial similarity with those of vertebrates, is organized differently. These results were confirmed by his embryological studies. Octopus rhabdomes, in fact, are quadratic like those of arthropods, and they are formed of four rhabdomeres from four different cells (Grenacher, 1884). The Hungarian anatomist Michael von Lenhossék described a simple layer of long palisade-like rods whose terminal part consists of a “Stäbchenspindel” (spindle region) filled with pigments. His splendid illustration and his scheme of the fine anatomy of the retina and the optic nerves of *Eledone* served as a model for many decades (Lenhossék, 1894). After World War II, John Zachary Young and his numerous collaborators resumed and refined the study of the cephalopod retina by applying electron microscopy (summarized in Young, 1971). The retinal ultrastructure was investigated also by Jerome J. Wolken, M. F. Moody, and J. R. Parriss, who demonstrated that the rhabdomere tubules show a dichroism and that the orientation of the rhodopsin molecules is geometric, thus providing a plausible explanation for the sensitivity of octopods to polarized light (Wolken, 1958; Moody and Robertson, 1960; Moody and Parris, 1961; Young, 1962).

The functioning of the photoreceptors aroused particular interest. Rawitz (1891) demonstrated that the pigments of the octopus eye migrate from an inner to an outer layer, and vice versa, when exposed to different conditions of illumination. Carl von Hess confirmed this movement (Hess, 1905). In 1902, he was the first to detect rhodopsin in *Loligo*, thus demonstrating that it is not exclusive to vertebrates. Yet, he guessed that its physiological behavior is different (Hess, 1902). Hess's idea that the level of pigment metabolism is of great importance in order to understand the process of phototransduction was soon confirmed by Bauer (1911). However, more than half a century passed before his intuition about rhodopsin conversion was confirmed by Paul and Patricia Brown, who provided biochemical proof that in *Octopus* and *Sepia* the rhodopsin produces a stable metarhodopsin (Brown and Brown, 1958).

Despite the uniqueness of the visual apparatus of cephalopods, great expectations were raised by the opportunity to transform them into experimental animals for the general understanding of the process of vision in camera-like eyes. Taking advantage of the neat arrangement of the eye's elements and the optic nerves, Adolf Beck succeeded in inquiring receptor sensitivity, obtaining simple response curves on exposure to light flashes for *Eledone* (Beck, 1899). Repeating Beck's work, a few years later, Hans Piper was the first to succeed in measuring the magnitude of the retinal electric response of *Eledone alta* (Piper, 1904). Cephalopods became definitively established as experimental objects for the electrophysiological research of vision when, in 1913, Friedrich Wilhelm Fröhlich obtained the first electroretinogram (ERG) with isolated *Eledone* and *Octopus* eyes (Fröhlich, 1913a,b). About half a century later, Brian Boycott resumed this Neapolitan research tradition and obtained electroretinograms in living and intact animals (Boycott et al., 1965). These successes raised concrete hopes that for the first time insights could be gained into the functioning of a complex neural and sensory system, inducing Stuart Sutherland, W.R.A. Muntz, N.J. Mackintosh and other psychologists to use *Octopus* to elaborate models of “visual pattern recognition” and the neurophysiological bases of learning (Sutherland, 1954; Sutherland and Muntz, 1959; Sutherland and Mackintosh, 1971).

## Color vision in cephalopods

Between 1909 and 1914, parts of one of the most famous disputes on whether animals are able to perceive and discriminate colors took place at Dohrn's Station. It started with fish, then switched to cephalopods—a still largely unknown episode—and finally to honeybees. Its protagonists—the then already established ophthalmologist Carl von Hess (1863–1923) and the then still unknown zoologist Karl von Frisch (1886–1982)—followed profoundly different approaches, so that the debate was transformed into more than just a scientific dispute (Autrum, 1963, 1990; Dröscher, 2005).

Hess's greatest achievement was the devising of a first reliable experimental system with which to study color discrimination and its application to a broad range of animal classes. In 1902 he came to Naples for the first time, in order to investigate the anatomy and physiology of the cephalopod eye (Hess, 1905), in particular rhodopsin, the pigment called “Sehpurpur” (visual purple) back then (Hess, 1902). Four years later, he made the “first attempt to systematically reveal how fish see” (Hess, 1909). For this purpose, he modified a technique, developed in Naples by Werner Krause, recording the reaction of *Amphioxus* in a tank exposed to lights of different brightness (Krause, 1897). Observing that in a dark room the fish *Atherina hepsetus* always swims toward the brightest part of the aquarium, Hess exposed them to monochromatic lights, and noted that their behavior resembled that of achromatopsic (colorblind) humans, when asked to move toward the brightest place in the room.

In order to investigate the color-brightness interaction, Hess then put *Atherina* in aquaria illuminated at one side by white light and by a certain color light at the other. Gradually modifying the brightness of the white light, he determined the exact moment when the fish stopped showing any preference. Again, the resulting graph turned out to be almost perfectly identical to the one obtained with achromatopsic humans. Hess concluded that fish are unable to distinguish different colors; rather, they react only to brightness (Hess, 1909, 1910c, 1912a). Extending his research to other vertebrate and invertebrate species (Hess, 1910a,b), he summarized his results in his famous monograph *Vergleichende Physiologie des Gesichtssinnes* (Hess, 1912b) establishing the by then dominating paradigm of the colorblindness of fish.

The strongest attack against Hess's results and his entire experimental system came from Karl von Frisch. Because Frisch was a zoologist and naturalist, he approached the question from a different standpoint. He considered the coincidence between the behavior of fish and achromatopsic humans to be a mere analogy. In this doctoral thesis he had investigated the control of body coloration and the chromatic matching of fish to the background (Frisch, 1910, 1911a, 1912b,c, 1913a). Then traveling to Naples, he experimented with the matching behavior of *Phoxinus laevis*. By varying the color of the background, he showed that the body coloration reaction differed even if the two colors had the same level of brightness (Frisch, 1911b). Frisch then devised learning experiments in which he trained the fish to react to saffron yellow. Thus, he created an association of a reward with a certain color. When exposed to little yellow cards stuck on a greater gray card having the same brightness, the fish reacted equally to the yellow cards (Frisch, 1912a). For Frisch this was proof that they were able to discriminate objects on the basis of their chromatic difference.

Before it reached its climax with the dispute on color vision in honeybees (Frisch, 1913b; Hess, 1913; Frisch, 1915; Menzel and Backhaus, 1989; Munz, 2016, pp. 32–50), the polemic between Hess and Frisch passed through a partially unknown episode that regarded cephalopods. Hess assumed their colorblindness. Unable to train them to swim toward lights, as he had done with fish, he had to develop a new experimental set-up. Some years previously, Rudolf Magnus had worked in Naples on the pupillary reaction of octopods, discovering that the closure of the eyelid is accompanied by a dilation of the pupil (Magnus, 1902). He also demonstrated that the pupillary reflex is not spontaneous but controlled by two distinct centers in the central ganglia. Based on these findings, Hess exposed the animals to lights of different colors and measured their pupillary reflex (*Sepia*) or their phototactic response (*Loligo*) in a tank so small that they could move only slightly forwards or backwards when trying to avoid the most disturbing lights. Again he noted a correspondence between the responses of cephalopods and achromatopsic humans (Hess, 1912b, pp. 331–345).

A few years later, Frisch again set out to contest Hess's results. On January 14, 1913 he wrote a letter to Reinhard Dohrn, ordering several marine species for his next stay at the Stazione, among them cephalopods. He revealed that he wanted “to train the animals to certain colors, in order to see with what other colors or gray papers they confound the color they had been trained for, a method very successfully applied to bees.” He then explained that he intended to train them,

“making double-walled test tubes, with colored paper between the tubes that are then fused in order to obtain colored, water-proof glass tubes. Then one feeds cephalopods several times a day (is this possible?) e.g., always with a crab leg, put inside the red test tube (obviously in a way that the animal does not see it) and shows him contemporaneously several differently colored tubes, the others are empty, so that it learns that only in the red one it will find something. Then, later, one shows it an empty red tube, instead of one filled with food, in order to see if it has learned to discriminate the colors and to see with which gray or colored papers it confounds the red, a procedure easy to manage with an appropriate positioning (Reinhard, 1914)^2^.”

Frisch never published his results. Consequently, we do not know if he actually carried out these experiments and how successful they were. Octopods show a great capacity of learning. Therefore, it is possible that Frisch performed them but that he did not obtain the desired results, and that he did not publish them, because his controversy with Hess had already reached a point where none of them could admit a failure. In fact, not only the results opposed Hess and Frisch. Their polemic was based on profoundly different approaches. Hess applied ophthalmological techniques, whereas Frisch acted as a naturalist. In Hess's sophisticated experimental system the animals were kept in precisely the conditions required to display the desired reactions, whereas Frisch tried to keep them in an environment that was as natural as possible. Frisch did this because he wanted to pose biologically meaningful questions, namely the adaptation of the animal's body color to the background or feeding preference. Hess, on the other hand, acted as an experimentalist, measuring reactions and drawing reductionist conclusions. The fact that, in the long run, Frisch's biological approach was awarded the Nobel Prize should not obscure that both ignored the role of the specific context in which their experimental objects displayed their behavior (Menzel and Backhaus, 1989). By placing the fish in a completely dark tank with sudden flashlights, Hess had created an emergency situation in which the animals did not care about colors and just swam toward the possible rescue, that is the brighter light. In Frisch's aquaria, instead, the animals were not fearing for their lives and had all the time necessary to make more nuanced choices.

Far from being definitively settled, the dispute on color vision continued to concern other researchers, who tried different experimental approaches. Based on his electroretinograms, Fröhlich demonstrated that octopods' retina reacted differently to different colors and brightness, and interpreted these responses as “the physiological basis of color discrimination.” For Fröhlich, *Octopus* was able to distinguish among red, yellow, green, and blue (Fröhlich, 1913b). The Dutch animal psychologist J.A Bierens de Haan failed in his attempts to train *Octopus* to discriminate colors (Bierens de Haan, 1926). Alfred Kühn, instead, hit *Octopus* with a stick after three brief monochromatic flashlights until the animal had learned to respond with an immediate flight, as soon as it perceived the colored light. When the octopod was then exposed to flashlights of another color but the same brightness, it did not flee, and Kühn deduced that it was able to distinguish colors (Kühn, 1930, 1950). Finally, between 1973 and 1977, John B. Messenger demonstrated with still other learning experiments that *Octopus* does not distinguish different colors. The animals were successfully trained to discriminate between rectangles differing in brightness, but failed to give the same response to rectangles differing in hue (Messenger et al., 1973; Messenger, 1977). However, octopods recognize the plane of polarized light, as John Z. Young had assumed on the basis of his studies on the geometry of octopus rhabdomeres (Young, 1960), a hypothesis then experimentally confirmed by Moody and Parris (1961).

Over the last 150 years, research on cephalopod vision has yielded many path-breaking specific and general insights, yet it has also shown that the initial expectation that it would be possible to understand how animals see, hear and feel, was vain and misleading. Today, less ambitious goals and more pragmatic definitions prevail (Kelber and Osorio, 2010).

## Author contributions

The author confirms being the sole contributor of this work and approved it for publication.

### Conflict of interest statement

The author declares that the research was conducted in the absence of any commercial or financial relationships that could be construed as a potential conflict of interest.

## Footnotes

^1^For a more complete bibliography see Ponte et al. (2013); and http://www.cephalopodresearch.org/cephs-science-history

^2^Letter from Karl von Frisch to Reinhard Dohrn, January 14, 1914, Historical Archives, Stazione Zoologica Anton Dohrn (Classified as Frisch 2013.A).
